# Nutrient Enrichment Coupled with Sedimentation Favors Sea Anemones over Corals

**DOI:** 10.1371/journal.pone.0125175

**Published:** 2015-04-21

**Authors:** Pi-Jen Liu, Min-Chieh Hsin, Yen-Hsun Huang, Tung-Yung Fan, Pei-Jie Meng, Chung-Cheng Lu, Hsing-Juh Lin

**Affiliations:** 1 Graduate Institute of Marine Biology, National Dong Hwa University, Pingtung, 944, Taiwan; 2 National Museum of Marine Biology and Aquarium, Pingtung, 944, Taiwan; 3 Department of Life Sciences and Research Center for Global Change Biology, National Chung Hsing University, Taichung, 402, Taiwan; 4 Biodiversity Research Center, Academia Sinica, Taipei, 115, Taiwan; University of Auckland, NEW ZEALAND

## Abstract

Fine sediments, which account for the majority of total fluvial sediment flux, have been suggested to degrade coral reefs on a global scale. Furthermore, sediment impacts can be exacerbated by extreme rainfall events associated with global climate change and anthropogenic nutrient enrichment. We report the findings from a series of mesocosm experiments exploring the effects of short-term sedimentation and nutrient enrichment on the interactions between the hard coral *Acropora muricata*, the sea anemone *Mesactinia ganesis*, and the green macroalga *Codium edule*. Mesocosms were manipulated to simulate either unimpacted reefs or reefs exposed to elevated levels of fine sediments for 10 or 14 days to simulate the effects of heavy rainfall. The first and second experiments were aimed to examine the effects of inorganic and organic sediments, respectively. The third experiment was designed to examine the interactive effects of nutrient enrichment and elevated sediment loads. Neither inorganic nor organic sediment loadings significantly affected the physiological performance of the coral, but, importantly, did reduce its ability to compete with other organisms. Photosynthetic efficiencies of both the green macroalga and the sea anemone increased in response to both sediment loadings when they were simultaneously exposed to nutrient enrichment. While organic sediment loading increased the nitrogen content of the green macroalga in the first experiment, inorganic sediment loading increased its phosphorus content in the second experiment. The coral mortality due to sea anemones attack was significantly greater upon exposure to enriched levels of organic sediments and nutrients. Our findings suggest that the combined effects of short-term sedimentation and nutrient enrichment could cause replacement of corals by sea anemones on certain coral reefs.

## Introduction

Amplification of the hydrological cycle as a consequence of global climate change has been forecasted to lead to more extreme levels of precipitation and thus surface runoff in many parts of the world [1]. During heavy rainfall episodes, streams and rivers often carry substantial sediment loads (originating mostly from landslides and stream bank erosion), and such events may become more commonplace in the near future. As such, there is a particular interest in understanding how sediment loading affects the structure and function of coastal ecosystems. Fine sediments, which are comprised predominantly of silts and clays, have been suggested to degrade coral reefs [2–3]; specifically, deposition of fine sediments has been shown to reduce coral growth, prevent recruitment and development of larvae, and change growth morphologies [4]. Fine sediments may become resuspended, and these suspended sediments may directly damage coral by abrasion, or, indirectly via decreasing light levels. Such a decrease in light may then result in decreased rates of photosynthesis and ultimately coral growth [5–6]. Although a significant body of research has been conducted on the effects of sedimentation on coral physiology, few studies have examined the effects of sedimentation on other coral reef inhabitants and consequent changes in species interactions. This represents a significant knowledge gap, particularly since sedimentation is recognized as a growing problem worldwide [7].

Nanwan Bay is located at the southern tip of Taiwan (21°57’N, 120°45’E) and is within Kenting National Park. It is a semi-enclosed embayment bounded by two capes, and there are well-developed fringing reefs distributed along the shoreline. In Taiwan, as elsewhere in the west Pacific Rim, intense precipitation, combined with high tectonic rates, drive rapid mass wasting and fluvial sediment transfer. However, the Nanwan Bay area has also suffered from extensive coastline development [8], and this has resulted in excessive sediment loads in certain places; indeed high turbidity levels can now be documented one to two weeks after a heavy rainfall event. Dense thickets of scleractinian corals of the genus *Acropora* were formerly dominant in the coral reefs of Nanwan Bay [9]. Over the past 15 years, however, many *Acropora* colonies have been lost. Sewage, sedimentation, and tourist impacts may all have decreased the resilience of *Acropora* colonies over this period. Additionally, a “super typhoon”and mass coral bleaching event in 1997 caused extensive *Acropora* death and reduced the ability of the survivors to compete with other sessile organisms, such as macroalgae and sea anemones. The macroalgae, most notably *Codium edule*, typically begin growing in response to sewage run-off and overfishing [8] and then compete with the coral for space or nutrients. If such conditions persist for several months, the macroalgae can overgrow and kill the corals [10].

At other times, the habitats of these *Acropora* colonies have been replaced by endosymbiotic sea anemones such as *Mesactinia genesis* and *Condylactis* sp. [11]. These sea anemones reproduce asexually year-round, and in 2003, sea anemone cover reached 50% in some patch reefs of Nanwan Bay [12]. Sea anemone outbreaks have been reported on coral reefs in Hawaii, Malaysia, and the Red Sea [13–15], but the causes are still unknown.

It has been assumed that typhoons, mass coral bleaching events, sewage run-off, sedimentation, and tourist impacts are the main factors contributing to loss of Nanwan Bay's *Acropora* meadows. As mentioned above, these seawater quality changes may have led to *C*. *edule* and sea anemone blooms, both of which are likely to further contribute to *Acropora* mortality [16–17]. Herein a series of mesocosm experiments were conducted to examine the interactions between corals (*Acropora muricata*), macroalgae (*C*. *edule*), and sea anemones (*M*. *ganesis*) under conditions of either inorganic or organic sediment enrichment coupled with elevated nutrient levels. Multiple experiments were conducted to 1) test for the effects of elevated inorganic sediment loads (experiment 1), 2) test for the effects of elevated organic sediment loads (experiment 2), and 3) examine the effects of high nutrient levels and sediment loads (to simulate the conditions of heavy rainfall periods in Nanwan Bay) on the competitive interactions between these three species. Building on the field observations mentioned above, we hypothesized that increased sedimentation, particularly in concert with high nutrient concentrations, would lead to increases in macroalgae and sea anemone cover at the expense of the coral species. As most marine algae are known to be nitrogen limited, the abundance of the macroalgae was hypothesized to increase in nutrient-enriched mesocosms [10]. It was further hypothesized that increased levels of sedimentation would reduce the photosynthetic performance of the algae. Finally, the endosymbiotic dinoflagellate (*Symbiodinium* sp.) densities as well as their chlorophyll *a* content were hypothesized to increase in corals exposed to nutrient enrichment, though potentially decrease in response to sedimentation given the decreased light levels that may be associated with such sediment load increases.

## Materials and Methods

### Coral reef mesocosm facility

**Fig 1:**
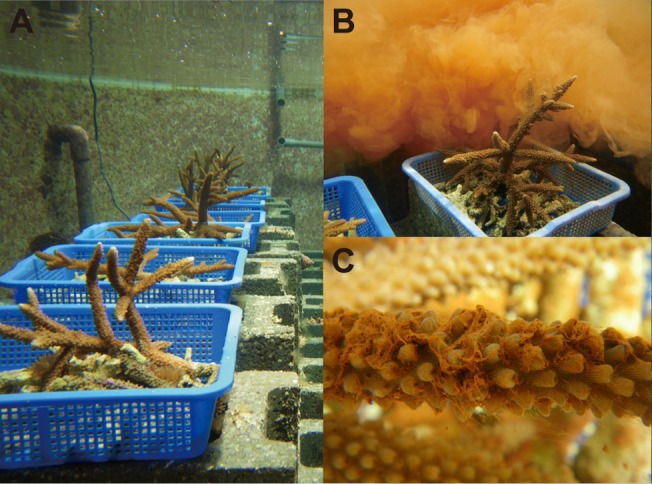
A coral reef mesocosm at the National Museum of Marine Biology and Aquarium, Southern Taiwan. Before (A) and during (B) sediment loading. A coral releasing mucus in response to sediment loading (C).

The Taiwan coral reef mesocosm facility is located at the National Museum of Marine Biology and Aquarium (NMMBA), approximately 10 km northwest of Nanwan Bay. Six mesocosms (5.14 m^2^ x 1 m depth) (Fig 1A) were designed to serve as living models of the fringing reefs of Nanwan Bay [10]. Flow-through seawater in the mesocosms was well-mixed by two pumps (120 L min^-1^) in order to produce a disturbance current that maintained fine sediments in suspension in the sediment-enriched mesocosms (described in more detail below). Sand filtered seawater pumped directly from adjacent Houwan Bay was added to each mesocosm at an exchange rate of 10% d^-1^ of the total volume. The exchanged seawater only flowed into these mesocosms for one hour every day before the sediment or nutrient loadings were conducted. During the exchange period, neither nutrients nor sediments were added to the experimental mesocosms. Water temperatures in the mesocosms were maintained between 25.0–26.0°C using a heat-exchanger to simulate field conditions in the Taiwanese spring [18]. The photosynthetically active radiation (PAR) at 0.5-m tank depth was maintained at 266 ± 5.0 *μ*mol photons m^-2^ s^-1^ from 0700 to 1700 hrs before sedimentation (a 10:14-h light: dark photoperiod) by metal halide lamps (OSRAM, HQI-BT 400 W/D).

The biological components (i.e., functional groups) of these mesocosms were cultured at the NMMBA for several years prior to experimentation. A detailed list of the organisms in each mesocosm can be found in S1 Table. The collection permits for the target coral *A*. *muricata*, the target macroalga, *C*. *edule*, and the target sea anemone *M*. *genesis* were issued by the Kenting National Park Headquarters before the study. Specimens of *A*. *muricata* (~15 cm diameter) and *M*. *genesis* were collected from 0.5 to 3.0 m depth in the inlet of the third Nuclear Power Plant (21°57’N, 120°45’E), where the average hourly PAR at 3-m depth was previously found to be 354 ± 63 *μ*mol photons m^-2^ s^-1^ [19]. *C*. *edule* (150 g wet weight per mesocosm) was collected from Nanwan Bay (21°57’N, 120°45’E) and Houwan Bay (22°02’N, 120°41’E).

### Experimental design

Three experiments were carried out between March and August 2009. In the first experiment, which was designed to document the effects of inorganic sediment (IS) loading alone (Exp. 1), three mesocosms were assigned to serve as controls (C), while the other three were enriched with IS. The sediments for all experiments were obtained by drying water collected downstream of Baoli Stream (N22°03'08'' W120°43'10'', no permits required) that had been sieved through a 61-*μ*m mesh. The dried sediments were then incinerated at 400°C in an oven to burn off the organic constituents. As the Nephelometric Turbidity Units (NTU) levels were found to reduce to half of their initial value eight hours after sediment addition (were no additional sediments added), in preliminary studies, 300 and 150 g of IS were added in the morning and afternoon, respectively (Fig 1B), in order to maintain the turbidity between 10–15 NTU at all times [20]. IS were added for 10 days, and the experiment was conducted for an additional 9 days after cessation of sediment enrichment to assess the effects of a recovery period.

In the second experiment (i.e., Exp. 2), which was designed to assess the effects of organic sediments (OS) alone, three mesocosms were assigned to serve as controls (C), while the other three were enriched with 200 g of OS twice daily, once in the morning and once in the afternoon. The OS were sieved through a 61-*μ*m mesh (without incineration) and added for 14 days. After cessation of the dosing, the experiment then ran for a further 14 days to assess the effects of a recovery period. In the third experiment (i.e., Exp. 3), which was designed to document the combined effects of sedimentation and nutrient loading, all six mesocosms were enriched with NaNO_3_ (5.50 mmol NO_3_ m^-2^ d^-1^) and KH_2_PO_4_ (0.48 mmol PO_4_^3^ m^-2^ d^-1^). This was equivalent to an N:P molar ratio of 12, which was intended to mimic anthropogenic nutrient inputs to coral reefs in Nanwan Bay [21]. Three of the six mesocosms were then enriched with OS as in Exp. 2. After 7 days of nutrient addition, OS were added as above for 14 days, and after 21 days of total experimental time, the systems were allowed to recover for 14 days after cessation of the experimental dosing.

In all experiments, turbidity, water temperature, salinity, pH, dissolved oxygen (DO) concentration, and PAR were monitored daily in each mesocosm as in [22]. Chlorophyll *a* concentrations in the water column were determined with a spectrophotometer by immediately filtering water samples in triplicate through Whatman GF/F filters and then extracting them in 90% acetone for 24 h at 4°C in the dark [23]. Water samples for nutrient analyses were collected and then filtered through Whatman GF/F filters, and concentrations of NO_3_, NO_2_, NH_3_, and PO_4_ were determined colorimetrically by a flow injection analytical method [24–25].

### Response variables

In order not to disturb the interactions among the macroalgae, sea anemones, and corals during the experimental period, changes in the abundance of the macroalga and coral were assessed by measuring the vertical projected cover from photographs taken throughout the experimental period with Image-Pro Plus 4.5 software (Media Cybernetics, Silver Spring, USA). The coral mortality was estimated by analysis of two-dimensional images of coral cover taken throughout the experiment. The sea anemone density was quantified by direct visual counting before and after each experiment.

The photosynthetic efficiencies of the macroalgae, as well as the *Symbiodinium* populations with the corals and sea anemones were approximated by measuring the maximum quantum yield of photosystem II (PS II) using a submersible pulse amplitude-modulated (Diving-PAM) fluorometer (Waltz). The fluorescence parameters of *F*_*o*_ (initial chlorophyll fluorescence after acclimating the specimens in darkness for 20 min when all reaction centers are open), *F*_*m*_ (maximum chlorophyll fluorescence after dark acclimation for 20 min when all reaction centers are closed following a saturating flash of light), and *F*_*v*_:*F*_*m*_ (maximum quantum yield of PS II, where *F*_*v*_ = *F*_*m*_—*F*_*o*_) were measured.

In order to analyze the chlorophyll *a* concentration, endosymbiotic *Symbiodinium* density, and protein content, a 3 cm branch of coral was collected before (2 days before sediment enrichment in Exps. 1 and 2; 3 days before nutrient enrichment or 10 days before sediment enrichment in Exp. 3), during (1 to 2 days after cessation of sediment enrichment in all three experiments), and after (9 and 17 days after cessation of sediment enrichment in Exps. 1 and 2, respectively; 14 days after cessation of sediments enrichment [nutrient levels remained enriched throughout this period]) each experiment. The surface area of each coral specimen was measured using the wax method [26] after coral tissues had been removed with a water jet [27]. The *Symbiodinium* density was quantified with a hemocytometer under a light microscope and normalized to surface area to yield cells cm^-2^. The chlorophyll *a* concentration [28] and protein content [29] were determined with a spectrophotometer and normalized to surface area.

Previous works [30–32] have found that nutrient enrichment can lead to differences in algal nitrogen and phosphorus content. Therefore, the C, N, and P concentrations of the macroalga were measured herein, and the latter two elements were expected to increase in concentration after a multi-week exposure to elevated nutrient levels. About 10 g (wet weight) of macroalga were collected after each experiment, dried at 60°C, and ground in a mortar for tissue C, N, and P analyses. Tissue C and N content were determined with a CHN-OS rapid element analyzer (Heraeus, Germany) according to the manufacturer’s recommendations. Tissue P content was measured colorimetrically with a spectrophotometer following persulfate digestion of the sample as in [33].

### Statistical analyses

A repeated-measures analysis of variance (ANOVA) was employed to determine whether the effects of time, treatments, or their interactions in each experiment had a significant effect on *Fv*:*Fm* with SAS (v9.1.3). Student’s *t*-tests were used to perform treatment comparisons between environmental (i.e., abiotic) and physiological response variables assessed before, during, and after each experiment. One-way ANOVAs were employed to perform experimental comparisons across treatments. Fisher’s least significant differences (LSD) tests were used for *post-hoc* means comparisons [34].

## Results

During the study period, water temperature was maintained at 25±0.5°C and salinity was 33±0.1 in all mesocosms. Turbidity and the level of suspended solids were significantly higher in the sediment-enriched mesocosms for all three experiments (Table 1). Conversely, DO concentrations and pH were significantly lower in the sediment-enriched mesocosms in all three experiments (Table 1). Chlorophyll *a* concentration in the water column was significantly lower in the sediment-enriched mesocosms of Exps. 1 and 2 (Table 1). However, the chlorophyll *a* concentration was higher in the nutrient-enriched mesocosms of Exp. 3, regardless of sediment loading. In Exps. 1 and 2, NO_3_＋NO_2_ and PO_4_ concentrations were within the normal range of the offshore waters of Nanwan Bay [35] and showed no significant differences between the control and sediment-enriched mesocosms at any sampling time (Table 1). However, the NO_3_＋NO_2_ concentration was significantly lower in IS-enriched mesocosms in Exp. 1. Furthermore, in Exp. 3, NO_3_＋NO_2_ and PO_4_ concentrations in the nutrient-enriched mesocosms increased about 2–6-fold relative to those in the mesocosms of Exps. 1 and 2, regardless of sediment loading.

**Table 1:** Physical and chemical characteristics of the coral reef mesocosms before, during, and after experiments.

	****Exp. 1****		****Exp. 2****		****Exp. 3****	
	****C1****	****IS****	****C2****	****OS****	****EC****	****ES****
**Photosynthetically active radiation (*μ*mol m** ^**-2**^ **s** ^**-1**^ **)**	112.4±7.6	85.0±7.4	115.8±2.0	67.2±1.8	125.7±5.2	67.9±4.3
**Dissolved oxygen (mg L** ^**-1**^ **)**						
Before	nd	nd	nd	nd	9.01±0.14	8.69±0.23
During	8.14±0.00	7.59±0.00***	8.18±0.12	7.17±0.10**	9.24±0.13	8.18±0.16**
After	8.21±0.02	7.90±0.03**	7.97±0.12	7.29±0.10*	9.22±0.22	8.79±0.08
**pH**						
Before	nd	nd	nd	nd	8.36±0.01	8.34±0.01
During	8.27±0.00	8.23±0.00***	8.25±0.02	8.13±0.00*	8.47±0.02	8.36±0.01*
After	8.31±0.01	8.24±0.01**	8.28±0.03	8.25±0.00	8.55±0.02	8.52±0.01
**Turbidity (NTU)**						
Before	nd	nd	nd	nd	0.24±0.14	0.41±0.15
During	0.22±0.00	5.83±0.11***	0.21±0.01	9.87±0.25***	0.22±0.02	10.38±0.10***
After	0.14±0.01	0.34±0.02**	0.50±0.03	0.83±0.06*	0.29±0.02	0.46±0.04*
**Suspended solids (g L** ^**-1**^ **)**						
Before	nd	nd	nd	nd	0.004±0.002	0.002±0.001
During	0.030±0.000	0.045±0.000***	0.026±0.001	0.035±0.000***	0.027±0.000	0.038±0.001***
After	0.030±0.001	0.033±0.000**	0.018±0.000	0.021±0.000**	0.020±0.001	0.026±0.002*
**Chlorophyll *a* concentration (mg m** ^**-3**^ **)**	0.15±0.01	0.12±0.01*	0.16±0.01	0.11±0.01**	0.27±0.02	0.27±0.01
**NO**_**3**_ **＋NO**_**2**_ **(*μ*M)**						
Before	0.34±0.02	0.39±0.06	1.28±0.20	1.37±0.21	0.77±0.06	0.86±0.21
During	0.76±0.05	0.49±0.04*	1.16±0.04	1.37±0.25	2.97±0.37	2.74±0.32
After	0.42±0.09	0.32±0.02	1.73±0.13	1.59±0.17	3.38±0.27	3.29±0.23
**PO**_**4**_ **(*μ*M)**						
Before	0.07±0.01	0.06±0.00	0.12±0.01	0.14±0.02	nd	nd
During	0.33±0.05	0.21±0.02	0.16±0.02	0.14±0.02	0.58±0.03	0.64±0.05
After	0.05±0.00	0.07±0.02	0.29±0.03	0.30±0.02	0.97±0.04	1.18±0.07
**NH**_**3**_ **(*μ*M)**						
Before	0.71±0.02	0.71±0.07	0.32±0.13	0.49±0.12	1.45±0.38	0.95±0.10
During	0.99±0.31	1.04±0.12	0.83±0.02	0.99±0.09	0.56±0.12	0.92±0.08
After	0.81±0.07	1.05±0.04	1.61±0.11	1.74±0.07	0.73±0.14	0.93±0.08

All values represent mean ± standard error (*n* = 3). Exp. 1: inorganic sediment enrichment, Exp. 2: organic sediment enrichment, Exp. 3: sediment and nutrient enrichment, *a priori* contrasts between control and treatment mesocosms were compared with student’s *t*-tests

*p < 0.05

**p < 0.01

***p < 0.001

C = control mesocosms, IS = inorganic sediment-enriched mesocosms, OS = organic sediment-enriched mesocosms, ES = nutrient and sediment-enriched mesocosms, EC = nutrient-enriched mesocosms, nd = not determined.

**Fig 2:**
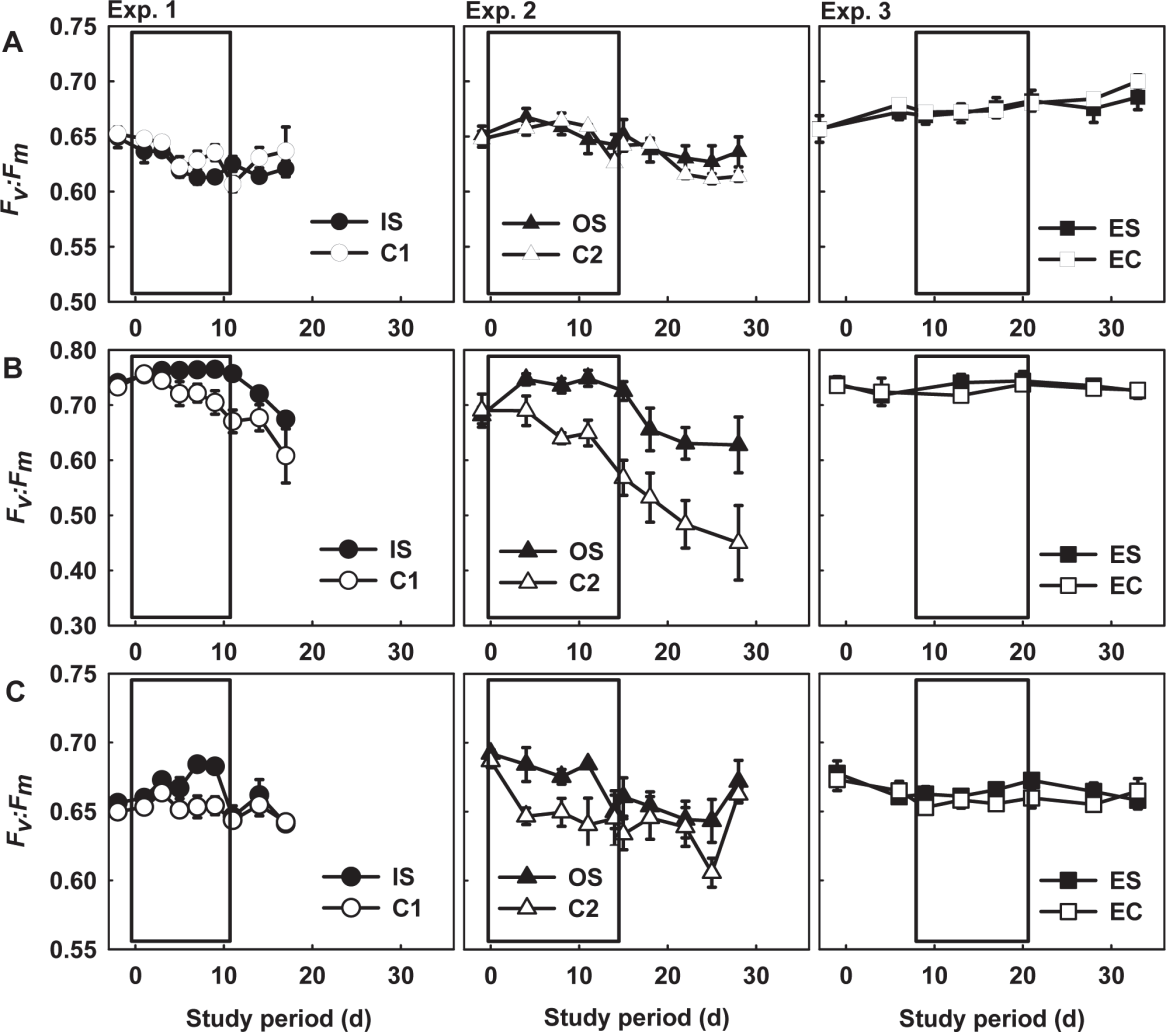
Changes in the maximum quantum yield of photosystem II. The *F*_*v*_:*F*_*m*_ (mean ± standard error [SE], *n* = 3) of the scleractinian coral *Acropora muricata* (A), the green macroalga *Codium edule* (B), and the sea anemone *Mesactinia genesis* (C). C = control mesocosms, IS = inorganic sediment-enriched mesocosms, OS = organic sediment-enriched mesocosms, ES = nutrient and sediment-enriched mesocosms, EC = nutrient-enriched mesocosms. The white box in the figure represents the sedimentation period.

In Exps. 1 and 2, the corals were observed to release mucus in response to increased inorganic and organic sedimentation (Fig 1C). On the other hand, the maximum quantum yield of photosystem II (*F*_*v*_:*F*_*m*_), protein content, and chlorophyll *a* content of the corals did not differ between control and sediment-enriched mesocosms. The *Symbiodinium* density was significantly lower in the IS-enriched mesocosms relative to the control mesocosms in Exp. 1 (Fig 2A; Table 2).

**Table 2:** Response of the coral *Acropora muricata* and the sea anemone *Mesactinia genesis* to sediment-enriched or nutrient-enriched seawater.

	****Exp. 1****		****Exp. 2****		****Exp. 3****	
	****C1****	****IS****	****C2****	****OS****	****EC****	****ES****
***Acropora muricata***						
**Chlorophyll *a* concentration (*μ*g cm** ^**-2**^ **)**						
Before	4.13±0.68	nd	5.28±0.20	nd	7.89±0.32	nd
During	4.47±0.28	4.04±0.28	5.92±0.36	6.71±0.14	9.76±0.93	9.80±0.58
After	3.04±0.06	3.14±0.21	4.62±0.45	4.86±0.33	10.62±0.22	9.90±0.13*
***Symbiodinium* density (10** ^**6**^ **cells cm** ^**-2**^ **)**						
Before	1.31±0.07	nd	1.75±0.04	nd	1.68±0.11	nd
During	1.53±0.02	1.34±0.01***	1.70±0.14	1.90±0.00	2.58±0.26	2.59±0.12
After	1.16±0.02	1.29±0.10	1.32±0.14	1.34±0.08	2.75±0.11	2.59±0.05
**Protein content (mg cm** ^**-2**^ **)**						
Before	0.19±0.01	nd	0.19±0.01	nd	0.20±0.00	nd
During	0.18±0.00	0.19±0.01	0.21±0.01	0.19±0.01	0.24±0.00	0.25±0.00
After	0.18±0.00	0.18±0.00	0.19±0.01	0.20±0.00	0.23±0.01	0.23±0.01
***Mesactinia genesis* density (individuals m** ^**-2**^ **)**						
Before	5.00±0.00	5.00±0.00	5.00±0.00	5.00±0.00	6.00±0.00	6.00±0.00
After	5.50±0.14	5.78±0.20	5.11±0.05	5.17±0.08	6.33±0.08	6.67±0.21
**Coral mortality by sea anemone attack (%)**	18.6±6.1	24.7±2.0	14.4±3.7	20.6±6.5	14.8±3.0	32.6±3.0*

All values represent mean ± standard error (*n* = 3). Exp. 1: inorganic sediment enrichment, Exp. 2: organic sediment enrichment, Exp. 3: sediment and nutrient enrichment. *a priori* contrasts between control and treatment mesocosms were compared with student’s *t*-tests

*p < 0.05

**p < 0.01

***p < 0.001

C = control mesocosms, IS = inorganic sediment-enriched mesocosms, OS = organic sediment-enriched mesocosms, ES = nutrient and sediment-enriched mesocosms, EC = nutrient enriched mesocosms, nd = not determined.

**Fig 3:**
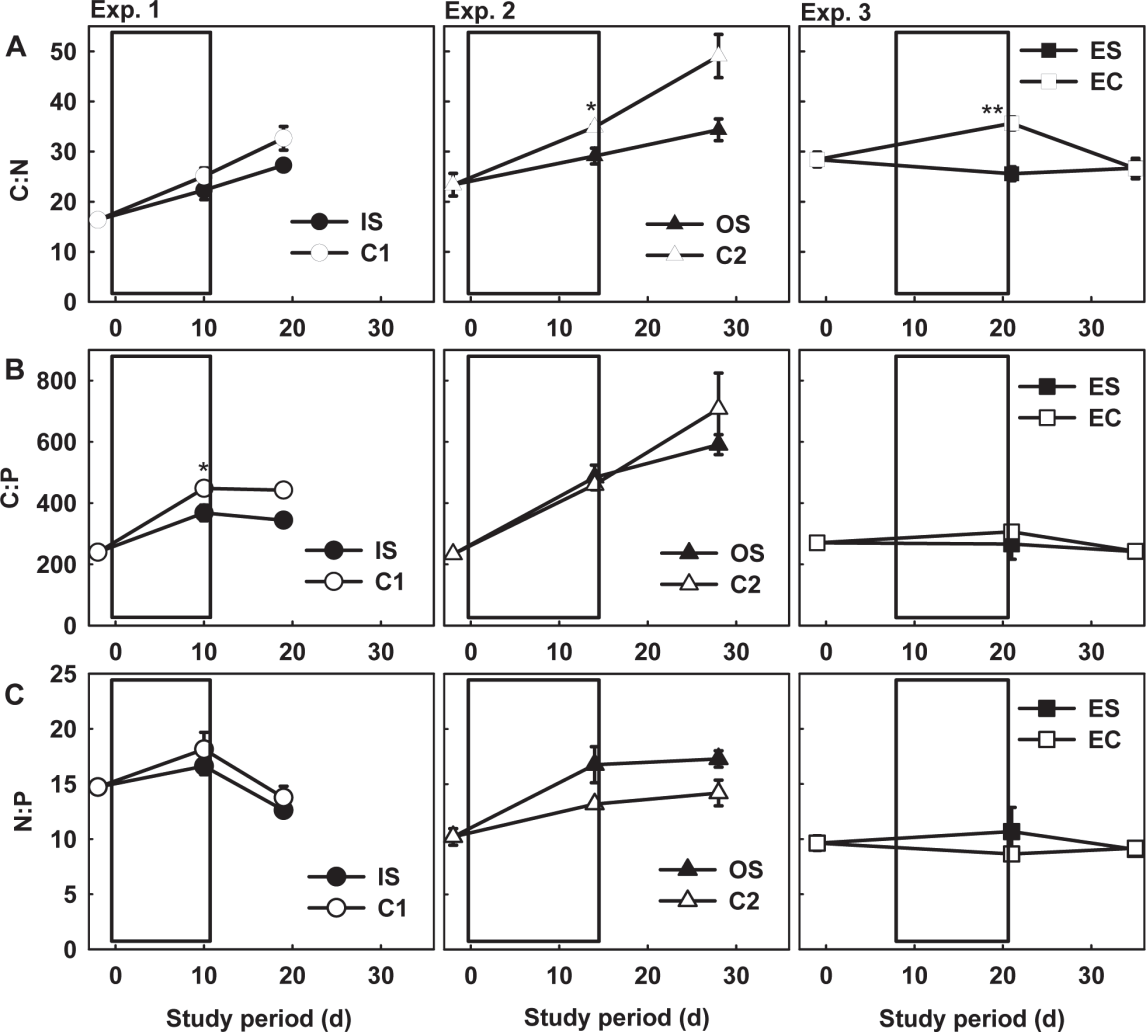
Changes in the tissue content of the green macroalga Codium edule. The C:N (A), C:P (B), and N:P molar ratios (C; mean ± standard error [SE], *n* = 3) of the green macroalga *Codium edule*. C = control mesocosms, IS = inorganic sediment-enriched mesocosms, OS = organic sediment-enriched mesocosms, ES = nutrient and sediment-enriched mesocosms, EC = nutrient-enriched mesocosms. The white box in the figure represents the sedimentation period.

Curiously, the *F*_*v*_:*F*_*m*_ values of *C*. *edule* slowly declined in the control mesocosms (Fig 2B). Furthermore, macroalgal *F*_*v*_:*F*_*m*_ values were significantly higher in the sediment-enriched mesocosms than in the controls during the sediment loading period (repeated-measures ANOVA: *F*_1,8_ = 25.77 and 20.57, *p* < 0.005). The *C*. *edule* C:N ratios in Exp. 2, as well as their C:P ratios in Exp. 1 in the sediment-enriched mesocosms were significantly lower than those of the control mesocosms (Fig 3A and 3B, respectively). However, the N:P ratios of the macroalgae showed no significant difference between the controls and sediment-enriched mesocosms (Fig 3C).

**Fig 4:**
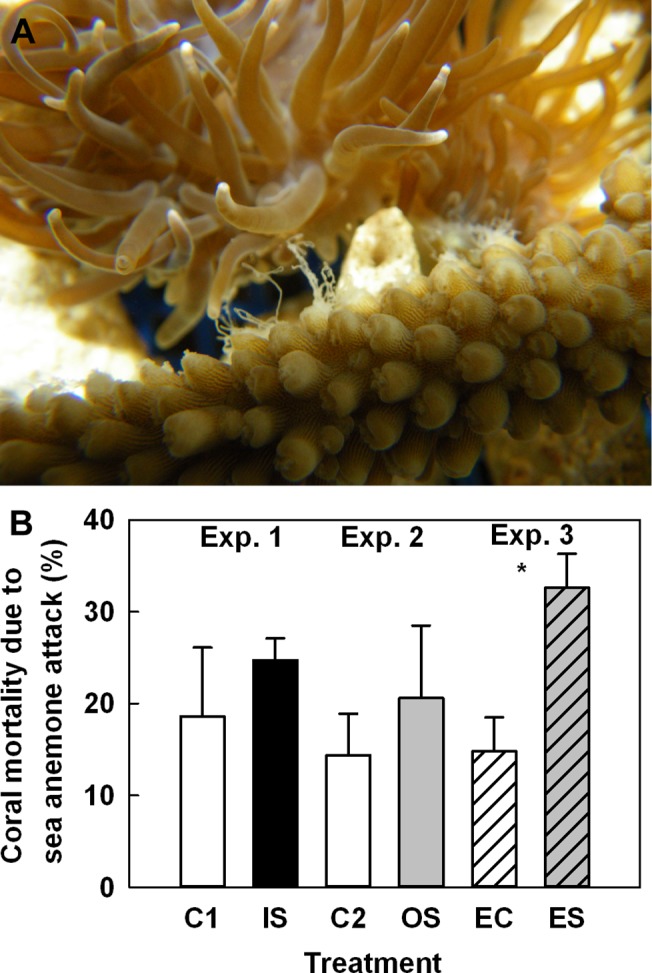
Sediment loading-induced inflation of modified attack tentacles of the sea anemone *Mesactinia genesis*. These attack tentacles (A) were capable of injuring or killing nearby corals. Coral mortality due to sea anemone attack (B). C = control mesocosms, IS = inorganic sediment-enriched mesocosms, OS = organic sediment-enriched mesocosms, ES = nutrient and sediment-enriched mesocosms, EC = nutrient-enriched mesocosms. Comparisons between control and experimental mesocosms were conducted with student’s *t*-tests (* *p* < 0.05).

The *F*_*v*_:*F*_*m*_ values of the sea anemones were significantly higher in the sediment-enriched mesocosms compared to the controls (Fig 2C, repeated-measures ANOVA: *F*_1,8_ = 10.42 and 5.65, *p* < 0.05). The sea anemone density did not increase in the sediment-enriched mesocosms (Table 2). Both IS and OS loadings stimulated the sea anemones to attack the coral with their attack tentacles (Fig 4A). However, the coral mortality due to sea anemone attack was not shown to differ significantly between treatments in either Exp. 1 or 2 (Table 2, Fig 4B).

In Exp. 3, the *F*_*v*_:*F*_*m*_ values of the corals, macroalgae, and sea anemones were consistently higher upon exposure to elevated nutrient concentrations, regardless of sediment loading (Fig 2, repeated-measures ANOVA: *F*_1,16_ = 0.31, 0.60, and 1.68, respectively, *p* > 0.05). The protein content, *Symbiodinium* density, and chlorophyll *a* concentration of the corals were also significantly higher under elevated nutrient exposure, regardless of sediment loading (Table 2). The C:N, C:P, and N:P ratios of the macroalgae were significantly lower after exposure to elevated nutrient levels in comparison to those in Exps. 1 and 2 (Fig 3, ANOVA: *F*_5,17_ = 10.8, 5.2, and 5.1 for C:N, C:P, and N:P ratios, respectively, *p* < 0.05 for all comparisons). Although the sea anemone density did not increase in nutrient-enriched mesocosms relative to densities in Exps. 1 and 2 (Table 2), the percent coral mortality attributed to attack by sea anemones was significantly greater (> 2-fold) under the combined effects of elevated sedimentation and elevated nutrients (Table 2, Fig 4B).

## Discussion

Our results revealed that *A*. *muricata* can tolerate sedimentation-driven water turbidity levels of up to 15 NTU for up to 14 days, a phenomenon that reduced irradiance by 30–50% (Table 1). This appears to be inconsistent with the general notion that sedimentation causes a decline in coral health [36], though it is possible that these corals may ultimately have succumbed to sedimentation stress had the experiment been carried out over a longer period of time. In this study, the corals were at a depth of 60 cm, and the PAR reaching the specimens remained at 68–85 *μ*mol m^-2^ s^-1^, even under heavy sediment loadings. This may explain the lack of an observed photoacclimation response of the coral to the relatively lower irradiance in the sediment-enriched mesocosms [18]. It appears, then, that the tolerance of corals to sedimentation-driven water turbidity increases may be dependent upon the light levels employed in the high turbidity treatments.

Corals have been observed to resist sedimentation by releasing mucus, vibrating and waving tentacles, or expanding their tissues, all of which require significant energy expenditures [37–39]. In this study, the insignificant effect of sediment loading on coral chlorophyll *a* concentration and *F*_*v*_:*F*_*m*_ could be partially attributable to the releasing of mucus to prevent smothering by fine sediments, which is similar to the observations of Anthony *et al*. [40]. However, this may lower the resistance of the corals to diseases caused by bacteria, cyanobacteria, fungi, and/or protozoan pathogens [41–42] or weaken their defenses against attack by other organisms. For instance, corals were observed to be killed by sea anemones at a higher frequency when sediment loading was coupled with nutrient enrichment (discussed in greater detail below).

The effects of inorganic and organic sediment loadings on the macroalga and the sea anemone were similar to the effects of nutrient enrichment observed on local coral reefs of Southern Taiwan [10]. In response to both IS and OS loadings, the *F*_*v*_:*F*_*m*_ values of the macroalga and sea anemone increased (Fig 2B and 2C). Although the sea anemone tended to increase its rate of asexual reproduction under elevated nutrient exposure, the percent mortality of corals due to sea anemone attack was significantly greater only under the combined effects of elevated sediments and nutrients (Table 2). The increased coral mortality may be attributed to the combined effects of lower competitive capacity in response to sediment loadings, given the potentially intensive energy expenditures such a situation would entail. On the other hand, the stimulation of asexual reproduction of sea anemones (i.e., the increase in anemone density noted in the Results) observed herein and in previous studies in response to nutrient enrichment [10] and exposure to elevated sediment levels in this study could have allowed the anemones to maintain a competitive advantage over the corals.

Although the concentrations of NO_3_＋NO_2_ and PO_4_ did not increase during the sediment loading experiments, the C:N and C:P ratios of the macroalgae were lower in the sediment-enriched mesocosms than in the controls, suggesting that N and P content were higher in the macroalgae exposed to high sediment loads. McGlathery *et al*. [31] and Birrell *et al*. [32] came to similar conclusions. The photosynthetic efficiency of the macroalgae was also higher in the sediment-enriched mesocosms than in the controls. It has been posed that macroalgae will maintain high photosynthetic efficiency under nutrient limitation, though not during periods of nutrient starvation [43–44]. We found that the photosynthetic efficiency of the macroalgae decreased in the control mesocosms herein (Fig 2B) and in previous studies [10], and the low nutrient concentrations may have accounted for this. In contrast, nutrients adsorbed to the IS or OS may have contributed to the higher *Fv*:*Fm* values of the macroalgae in the IS and OS-enriched mesocosms (Table 1).

The N:P ratios of the macroalgae showed some noteworthy differences between the IS and OS treatments; although the N:P ratios of the macroalgae tended to be lower in the IS-enriched mesocosms, they were higher in the OS-enriched mesocosms (Fig 3C). This suggests that relatively more P was available for the macroalgae receiving high IS loads, and relatively more N was available for the macroalgae receiving high OS loads. This is also evidenced by the fact that the N:P ratios of the macroalgae were significantly higher in the mesocosms receiving OS and nutrient enrichment than in the mesocosms receiving nutrient enrichment only (Exp. 3C). One explanation for the relatively high P content of the macroalgae exposed to high levels of IS could be due to having released P from the incineration of the sediments [45], particularly the fine silts.

Although sea anemones are widely considered to compete with corals and macroalgae for space [12], the competitive hierarchy between the two taxa has been understudied. This study provides direct, experimental evidence that these species of coral, macroalga, and sea anemone can coexist under ambient conditions. However, under nutrient- and sediment-enriched conditions, there was an active and aggressive interaction between the sea anemone and the coral, and, furthermore, an increase in proliferation of the former. Previous mesocosm experiments [10] suggested that the hierarchy of competitive superiority under nutrient enrichment was in the order of the macroalga > sea anemone > coral; in this study, however, the cover of the macroalga was low. Furthermore, *C*. *edule* was not observed to grow fast enough to smother the coral during experimental periods, although both their nutrient content and *F*_*v*_:*F*_*m*_ values increased.

In Nanwan Bay, *M*. *ganesis* is more frequently observed in the rainy season [16–17]. It appears that the sea anemone out-competes the macroalga for space at these times, although the sea anemone was observed to move away from fast-growing macroalgae in the nutrient-enriched mesocosms of another study [10]. In this study, the water temperature in the mesocosms was maintained at ~ 25°C, which is the water temperature at which spring *C*. *edule* blooms occur in Nanwan Bay [8]. A seasonal pattern of macroalgal abundance, in which greater biomass was observed in winter and spring and lower biomass was observed in summer and fall, has been documented in Southwestern Taiwan [46], and it has been suggested that high temperature may thwart their summer growth rates.

Therefore, *M*. *ganesis* is expected to respond to sedimentation under elevated nutrient levels *in situ* by potentially replacing *C*. *edule* in the summer months when water temperatures increase to over 28°C and southwest winds bring considerable quantities of rain [47], at which time the macroalgal abundance declines. In our experiments, which were conducted at seawater temperatures corresponding to those of the spring, the high sediment dosings may have provided nutrients for the macroalgae that allowed them maintain a high level of physiological performance even under the lower irradiances caused by the sedimentation.

The asexual reproduction rate of the sea anemones, which has been shown to be critical in other locations at which the replacement of corals by sea anemones has been documented to occur [48], was lower than rates calculated in previous studies in Southern Taiwan [10]. Furthermore, sedimentation is typically a short-term influence on coral reefs in Taiwan, and while sea anemones were found herein to kill and overgrow portions of coral colonies during periods of elevated nutrients and sedimentation, their growth rates alone cannot explain their ability to rapidly replace *Acropora* colonies *in situ*. Therefore, additional factors must be responsible for the near-total replacement of certain coral species by sea anemones that occurs *in situ* on certain reefs of Southern Taiwan.

In conclusion, the high physiological performance of the sea anemones and macroalgae, the potentially stressed state of the corals due to having had to expend considerable energy on mucus production, and the higher likelihood of sea anemone attacks against corals in the sediment and nutrient-enriched mesocosms suggest that such conditions *in situ* could lead to anemone overgrowth of corals. Future work should attempt to elucidate the additional abiotic or biotic parameters that act to shift this competitive balance *in situ*.

## Supporting Information

S1 Table The biological components (i.e., functional groups) of the experimental mesocosms. (DOC)

## References

[1] Intergovernmental Panel on Climate Change (2007) Climate Change 2007: Synthesis Report. Contribution of Working Groups I, II and III to the fourth assessment report of the Intergovernmental Panel on Climate Change. (eds Core writing team, Pachauri, R.K. & Reisinger, A.). Switzerland Press, Geneva. 104 p.

[2] Brodie JE, Christie C, Devlin M, Haynes D, Morris S, Ramsay M, et al (2001) Catchment management and the Great Barrier Reef. Water Sci Technol 43: 203–211. 11419129

[3] Devlin MJ, Brodie J (2005) Terrestrial discharge into the Great Barrier Reef Lagoon: nutrient behaviour in coastal waters. Mar Pollut Bull 51: 9–22. 15757704 10.1016/j.marpolbul.2004.10.037

[4] Syvitski JPM, Vörösmarty CJ, Kettner AJ, Green P (2005) Impact of humans on the flux of terrestrial sediment to the global coastal ocean. Science 308: 376–380. 15831750 10.1126/science.1109454

[5] Riegl B, Branch GM (1995) Effects of sediment on the energy budgets of four scleractinian (Bourne 1900) and five alcyonacean (Lamouroux 1816) corals. J Exp Mar Biol Ecol 186: 259–275.

[6] Philipp E, Fabricius K (2003) Photophysiological stress in scleractinian corals in response to short-term sedimentation. J Exp Mar Biol Ecol 287: 57–78.

[7] Golbuu Y, Victor S, Wolanski E, Richmond RH (2003) Trapping of fine sediment in a semi-enclosed bay, Palau, Micronesia. Estuar Coast Shelf S 57: 941–949.

[8] Dai CF (1997) Assessment of the present health of coral reefs in Taiwan In: Status of Coral Reefs in the Pacifi*c* (eds Grigg RW, Birkeland C). Univ of Hawaii Press, United States, pp. 123–131.

[9] Dai CF (1993) Patterns of coral distribution and benthic space partitioning on the fringing reefs of southern Taiwan. Mar Ecol 42: 185–204.

[10] Liu PJ, Lin SM, Fan TY, Meng PJ, Shao KT, Lin HJ (2009) Rates of overgrowth by macroalgae and attack by sea anemones are greater for live coral than dead coral under conditions of nutrient enrichment. Limnol Oceanogr 54: 1167–1175.

[11] Chen CA, Dai CF (2004) Local phase shift from *Acropora-*dominant to *Condylactis-*dominant community in the Tiao-Shi Reef, Kenting National Park, southern Taiwan. Coral Reefs 23: 508.

[12] Tkachenko KS, Wu BJ, Fang LS, Fan TY (2007) Dynamics of a coral reef community after mass mortality of branching *Acropora* corals and an outbreak of anemones. Mar Biol 151: 185–194.

[13] Cooke WJ (1976) Reproduction, growth and some tolerances of *Zoanthus pacificus* and *Palythoa vestitus* in Kaneohe Bay, Hawaii In: Coelenterate Ecology and Behavior (ed Mackie GO). Plenum Press, New York, pp. 281–288.

[14] Ridzwan AR (1993) Recovery processes of coral communities following the crown-of-thorns starfish, *Acanthaster planci*, infestations on the east coast islands of Peninsular Malaysia. PhD thesis Univ of Newcastle Press, United Kingdom. 303 p.

[15] Chadwick-Furman NE, Spiegel M (2000) Abundance and clonal replication in the tropical corallimorpharian *Rhodactis rhodostoma* . Invertebrate Biol 119: 351–360.

[16] Dai CF, Kao KM, Chen YT, Chaun ST (1998) Changes of coral communities in the Nan-wan Bay, Kenting National Park from 1987 to 1997. J Natl Park 8: 79–99. (in Chinese with English abstract)

[17] Dai CF, Kao KM, Chen YT, Chaun ST (1999) Changes of coral communities in the eastern and western coast, Kenting National Park from 1987 to 1997. J Natl Park 9: 111–129. (in Chinese with English abstract)

[18] Mayfield AB, Chan PH, Putnam HP, Chen CS, Fan TY (2012) The effects of a variable temperature regime on the physiology of the reef-building coral *Seriatopora hystrix*: results from a laboratory-based reciprocal transplant. J Exp Biol 215: 4183–4195. doi: 10.1242/jeb.071688 22933614 10.1242/jeb.071688

[19] Mayfield AB, Fan TY, Chen CS (2013) Physiological acclimation to elevated temperature in a reef-building coral from an upwelling environment. Coral Reefs 32: 909–921.

[20] Meng PJ, Chen JP, Chung KN, Liu MC, Fan TY, Chang CM, et al (2004) Long-term ecological studies in Kenting National Park neighboring marine areas, on monitoring the impact factors from anthropogenic activities to the marine ecosystem and a preliminary database of its marine ecosystem. J Natl Park 14: 43–69. (in Chinese with English abstract)

[21] Lin HJ, Wu CY, Kao SJ, Kao WY, Meng PJ (2007) Mapping anthropogenic nitrogen through point sources in coral reefs using δ15N in macroalgae. Mar Ecol Prog Ser 335: 95–109.

[22] Liu PJ, Meng PJ, Liu LL, Wang JT, Leu MY (2012) Impacts of human activities on coral reef ecosystems of southern Taiwan: A long-term study. Mar Poll Bull 64: 1129–1135. 10.1016/j.marpolbul.2012.03.031 22534409

[23] Parsons TR, Maita Y, Lalli CM (eds) (1984). A Manual of Chemical and Biological Methods for Seawater Analysis. Pergamon Press, New York. 173 p.

[24] Strickland JDH, Parsons TR (1972) A Practical Handbook of Seawater Analysis. Bull Fish Res Bd Can 167, 2nd ed, Ottawa. 310 p.

[25] Pai SC, Yang CC, Riley JP (1990) Formation kinetics of the pink azo dye in the determination of nitrite in natural waters. Anal Chim Acta 232: 345–349.

[26] Stimson J, Kinzie RA (1991) The temporal pattern and rate of release zooxanthellae from the reef coral *Pocillopora damicornis* (Linnaeus) under nitrogen-enrichment and control conditions. J Exp Mar Biol Ecol 153: 3–74.

[27] Szmant AM, Gassman NJ (1990) The effects of prolonged “bleaching” on the tissue biomass and reproduction of the reef coral *Montastrea annularis* . Coral Reefs 8: 217–224.

[28] Jeffery SW, Humphrey GF (1975) New spectrophotometric equations for determining chlorophylls a, b, c1, and c2 in higher plants, algae and natural phytoplankton. Biochem Physiol Pflanz 167: 191–194.

[29] Bradford M (1976) A rapid and sensitive method for the quantitation of microgram quantities of protein utilizing the principle of protein dye binding. Anal Biochem 72: 246–254. 10.1016/0003-2697(76)90527-3 942051

[30] Richardson CJ, Marshall PE (1986) Processes controlling movement, storage, and export of phosphorus in a fen peatland. Ecol Monogr 56: 279–302.

[31] McGlathery KJ, Krause-Jensen D, Rysgaard S, Christensen PB (1997) Patterns of ammonium uptake within dense mats of the filamentous macroalga *Chaetomorpha linum* . Aquat Bot 59: 99–115.

[32] Birrell CL, McCook LJ, Willis BL (2005) Effects of algal turfs and sediment on coral settlement. Mar Pollut Bull 51: 408–414. 15757739 10.1016/j.marpolbul.2004.10.022

[33] Solorzano L, Sharp JH (1980) Determination of total dissolved phosphorus and particulate phosphorus in natural waters. Limnol Oceanogr 25: 754–758.

[34] Sokal RR, Rohlf FJ (1995) Biometry: the Principals and Practice of Statistics in Biological Research, 3rd ed, WH Freeman and Co., New York. 887 p.

[35] Chen CC, Shiah FK, Lee HJ, Li KY, Meng PJ, Kao SJ, et al (2005) Phytoplankton and bacterioplankton biomass, production and turnover in a semi-enclosed embayment with spring tide induced upwelling. Mar Ecol Prog Ser 304: 91–100.

[36] Fabricius KE (2005) Effects of terrestrial runoff on the ecology of corals and coral reefs: review and synthesis. Mar Pollut Bull 50: 125–146. 15737355 10.1016/j.marpolbul.2004.11.028

[37] Bak RPM, Elgershuizen JHBW (1976) Patterns of oil-sediment rejection in corals. Mar Biol 37: 105–113.

[38] Stafford-Smith MG (1993) Sediment-rejection efficiency of 22 species of Australian scleractinian corals. Mar Biol 115: 229–243. 8054598

[39] Gilmour JP (2002) Acute sedimentation causes size-specific mortality and asexual budding in the mushroom coral, *Fungia fungites* . Mar Freshwater Res 53: 805–812.

[40] Anthony KRN, Ridd PV, Orpin AR, Larcombe P, Lough J (2004) Temporal variation of light availability in coastal benthic habitats: effects of clouds, turbidity and tides. Limnol Oceanogr 49: 2201–2211.

[41] Linton D, Smith R, Alcolado P, Hanson C, Edwards P, Estrada R, et al (2002) Status of coral reefs in the northern Caribbean and Atlantic node of the GCRMN In: Status of coral reefs of the world (ed Wilkinson CR). GCRMN Report, Australian Institute of Marine Science, Townsville, pp. 277–302.

[42] Sutherland KP, Porter JW, Torres C (2004) Disease and immunity in Caribbean and Indo-Pacific zooxanthellate corals. Mar Ecol Prog Ser 266: 273–302.

[43] Wiedenmann J, D’Angelo C, Smith EG, Hunt AN, Legiret F-E, Postle AD, et al (2013) Nutrient enrichment can increase the susceptibility of reef corals to bleaching. Nat Clim Change 3: 160–164.

[44] D’Angelo C, Wiedenmann J (2014) Impacts of nutrient enrichment on coral reefs: new perspectives and implications for coastal management and reef survival. Curr Opin Environ Sustainability 7: 82–93.

[45] Ketterings QM, van Noordwijk M, Bigham JM (2002) Soil phosphorus availability after slash-and-burn fires of different intensities in rubber agroforests in Sumatra, Indonesia. Agric Ecosyst Environ 92: 37–48.

[46] Lin HJ, Hung JJ (2004) Factors affecting macroalgal distribution in a eutrophic lagoon in Taiwan. Mar Biol 144: 653–664.

[47] Lin HJ, Shao KT (1998) Temporal changes in the abundance and growth of intertidal *Thalassia hemprichii* seagrass beds in southern Taiwan. Bot Bull Acad Sinica 39: 191–198.

[48] Wiedenmann J, Leutenegger A, Gundel S, Schmitt F, D’Angelo C, Funke W (2007) Long-term monitoring of space competition among fluorescent and nonfluorescent sea anemones in the Mediterranean Sea. J Mar Biol Assoc UK 87: 851–852.

