# Supplementary material for: Nutrient Enrichment Coupled with Sedimentation Favors Sea Anemones over Corals
Source: PLoS One. 2015 Apr 21;10(4):e0125175. doi: 10.1371/journal.pone.0125175 (PMC4405573; doi:10.1371/journal.pone.0125175)
Supplement: S1 Table — (DOC) [file pone.0125175.s001.doc]

**S1 Table. The biological components (i.e., functional groups) of the experimental mesocosms.**

|  |  | treatment | Control | | | Sediment-enriched | | |  |
| --- | --- | --- | --- | --- | --- | --- | --- | --- | --- |
| common name |  | tank | C1 | C2 | C3 | S1 | S2 | S3 | unit |
| Branching coral | *Acropora muricata* | | 3 | 3 | 3 | 3 | 3 | 3 | colony |
|  | *Montipora stellata* | | 3 | 3 | 3 | 3 | 3 | 3 | colony |
|  | *Pocillopora damicornis* | | 2 | 2 | 2 | 2 | 2 | 2 | colony |
|  | *Stylophora pistillata* | | 2 | 2 | 2 | 2 | 2 | 2 | colony |
| Massive coral | *Heliopora coerulea* | | 4 | 4 | 4 | 4 | 4 | 4 | colony |
| Macroalgae | *Padina minor* | | 23.34 | 22.67 | 24.47 | 27.09 | 29.55 | 22.41 | g |
|  | *Turbinaria ornate* | | 50.12 | 58.85 | 56.50 | 62.88 | 64.88 | 74.52 | g |
|  | *Hydroclathrus clathratus* | | 42.01 | 25.39 | 39.87 | 38.06 | 37.50 | 27.23 | g |
| Blackspot sergeant fish | *Abudefduf sordidus* | | 11.36 | 10.70 | 22.90 | 16.03 | 13.84 | 12.15 | g |
| Bowtie damselfish fish | *Neoglyphidodon melas* | | 3.34 | 3.15 | 3.48 | 3.56 | 4.36 | 2.60 | g |
| Giant clam | *Tridacna maxima* | | 2 | 2 | 2 | 2 | 2 | 2 | individual |
| Gold-ringed cowrie | *Cypraea annulus* | | 1 | 1 | 1 | 1 | 1 | 1 | individual |
| Sea snails | *Trochus hanleyanus* | | 20 | 20 | 20 | 20 | 20 | 20 | individual |
| Turban snails | Turbinid sp. | | 10 | 10 | 10 | 10 | 10 | 10 | individual |
| Hermit crab | Paguroidea sp. | | 2 | 2 | 2 | 2 | 2 | 2 | individual |
| Sea cucumbers | *Holothuria leucospiolota* | | 3 | 3 | 3 | 3 | 3 | 3 | individual |
| Sea urchins | *Tripneustes gratilla* | | 2 | 2 | 2 | 2 | 2 | 2 | individual |
